# The prognostic impact of C‐reactive protein and albumin in patients diagnosed with acute myeloid leukaemia

**DOI:** 10.1002/jha2.1022

**Published:** 2024-10-17

**Authors:** Espen Talseth Skar, Øystein Wendelbo, Håkon Reikvam

**Affiliations:** ^1^ Department of Clinical Science, University of Bergen K.G. Jebsen Center for Myeloid Blood Cancer Bergen Norway; ^2^ Department of Medicine Haukeland University Hospital Bergen Norway; ^3^ Department of Nursing Faculty of Health VID Specialized University Bergen Norway

**Keywords:** acute myeloid leukaemia, CRP‐albumin ratio, Glasgow Prognostic Score

## Abstract

**Background:**

Acute myeloid leukaemia (AML) is an aggressive and heterogeneous malignant disease. Patient age, comorbidities and disease‐specific genetic abnormalities are recognized as primary determinants of treatment response. Recent years have elucidated the significance of nutritional status and inflammation across various malignancies, including AML, in influencing treatment outcomes.

**Aims:**

To assess the prognostic value of the C‐reactive protein‐albumin ratio (CAR) and the Glasgow Prognostic Score (GPS) in predicting overall survival (OS) rates among patients diagnosed with AML.

**Material and methods:**

189 AML patients receiving standard cytarabine and anthracycline‐based induction treatment were included. Baseline demographic, clinical and laboratory data were collected, and treatment outcomes and survival were registered for all patients.

**Results:**

No significant association between CAR and prognosis among AML patients was found, even in subgroup analyses. Hypoalbuminemia was an independent predictor of poor survival among all patients (OS 28 vs. 16 months; *p* < 0.02). Patients with a GPS of 0 or 1 demonstrated superior OS compared to those with a GPS of 2 (median OS 28 vs. 16 months, respectively; *p* = 0.015). Results remained consistent among patients ≥ 60 years (median OS 15 vs. 6 months; *p* = 0.020).

**Conclusion:**

Heightened inflammation and suboptimal nutritional status correlate with unfavourable prognoses in AML patients. Such insights hold the potential for guiding clinical decision‐making, offering easily accessible prognostic information for the induction treatment of eligible AML patients.

## 1 INTRODUCTION

Acute myeloid leukaemia (AML) is the most common acute type of leukaemia. AML is a heterogeneous disease caused by clonal expansion of immature progenitor cells within the myeloid lineage, and although improvements have been made over the last years regarding treatment and risk stratification, accurately estimating prognosis remains challenging [1].

Risk assessment in AML incorporates disease‐related factors and patient‐related factors [2, 3]. Established patient‐related factors include age, performance status and comorbidities. Disease‐related factors associated with outcome include established molecular genetic mutations, especially the presence of FMS‐like tyrosine kinase 3 (*FLT3*) and nucleophosmin 1 (*NPM1*) gene mutations. The European Leukaemia Network (ELN) risk stratification model currently categorizes patients into favourable, intermediate and adverse risk groups [4].

Inflammation plays a pivotal role in the development and progression of malignant diseases [5, 6, 7]. Previous studies have documented an association between inflammation, reflected by serum levels of C‐reactive protein (CRP) and albumin, respectively; and prognosis [8, 9, 10, 11, 12, 13]. Furthermore, CRP to albumin ratio (CAR) has been elucidated to add prognostic value in AML patients [14]. CAR has been shown to be an independent prognostic factor for overall survival (OS) in AML patients of advanced age not eligible for allogeneic hematopoietic stem cell transplantation (allo‐HSCT) [15]. In addition, other inflammatory markers have been linked to prognosis in AML. Ferritin levels, which serve as both a marker of iron overload [16], although also inflammation and an indicator of genetic changes in AML tumour cells, have been shown to be prognostic at the time of diagnosis in patients eligible for chemotherapy [8, 11, 17–20]. Similarly, high fibrinogen levels have been associated with poorer prognosis in AML [21, 22].

The Glasgow Prognostic Score (GPS) and modified GPS (mGPS) are validated risk calculators based on CRP and albumin levels, demonstrated to predict survival in cancer patients, including gastric, lung and renal cancer [8, 10, 11, 17, 23–28] and for mGPS even in cancer patients independent of tumour site [29, 30].

We performed a study to evaluate the association between survival and the serum levels of CRP and albumin at the time of diagnosis in patients with AML receiving standard induction treatment.

## 2 PATIENT POPULATION AND METHODS

### 2.1 Ethical consent

The Regional Ethics Committee (REK Vest) classified the study as quality assurance (REK‐Vest number 404696); therefore, written consent was not considered necessary. The hospital data protection officer approved the study, and all identifiable patient information was stored on a secure server.

### 2.2 Patient selection

Using a digital search tool based on diagnosis codes stored in the electronic patient journal system, we extracted every patient with a registered AML diagnosis from 2001 to 2021. Patients under the age of 18 years at the time of diagnosis, and patients not receiving cytarabine and anthracycline‐based induction therapy were excluded from the study.

**FIGURE 1:**
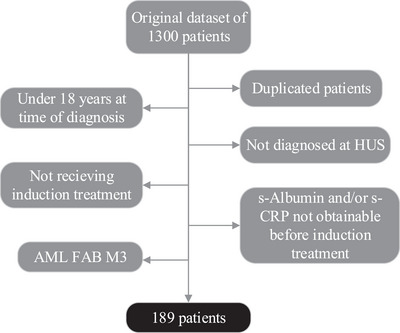
Patient selection among 1300 subjects resulted in a final study population of 189 AML patients aged 18 years or older at the time of diagnosis receiving induction treatment at Haukeland University Hospital between 2001 and 2021. Duplicated patients, patients younger than 18 years at the time of diagnosis, patients diagnosed or treated at a different centre than Haukeland University Hospital, patients receiving palliative care, patients with acute promyelocyte leukaemia and patients where CRP and/or albumin were not obtainable prior to induction treatment were removed. HUS: Haukeland University Hospital. CRP: C‐reactive protein. AML: acute myeloid leukaemia. FAB: French American British classification.

We initially obtained 1300 subjects with a diagnosis of AML. After removing duplicates, patients where laboratory serum albumin and/or serum CRP‐levels were not attainable within the start of induction treatment, patients with AML diagnosis or treatment sat in a different centre, and patients with acute promyelocytic leukaemia (APL), we were left with 189 patients (Figure 1).

### 2.3 Data extraction

We extracted data including French American British (FAB) subtype, blast count in peripheral blood and/or bone marrow, *NPM1* and *FLT3* mutation status, cytogenetic profile, remission status after induction treatment and transplantation status including autologous hematopoietic stem cell transplantation (auto‐HSCT) or allo‐HSCT. The laboratory data included haemoglobin (Hb), white blood cell count (WBC), thrombocyte count, CRP, albumin, lactate dehydrogenase (LDH) and creatinine. Laboratory data was obtained upon admission, and prior to induction therapy.

CAR was calculated by dividing CRP by albumin levels. By using the median CAR, patients were divided into either a low CAR (CAR < 0.82) or a high CAR (CAR ≥ 0.82). GPS and mGPS range from 0 to 2 based on CRP and albumin, where combinations of serum CRP ≥10/< 10 mg/L and/or serum albumin ≥35/<35 g/L dictate the score. GPS0 is defined as a CRP <10 mg/L and albumin ≥35g/L. GPS1 is defined as either increased CRP ≥10 mg/L with a normal albumin ≥35g/L or a normal CRP <10 mg/L with decreased albumin < 35 g/L. GPS2 is defined as both increased CRP ≥10 mg/L and decreased albumin <35 g/L [10]. The mGPS differs from the GPS by having more emphasis on inflammation. mGPS0 is defined as a CRP <10 mg/L independent of albumin value. mGPS1 is defined as increased CRP >10 mg/L with a normal albumin ≥35g/L and mGPS2 is equal to GPS2 [29].

### 2.4 Statistical analysis

Statistical analysis was performed by using GraphPad Prism version 10.0.0 for Mac (GraphPad Software). Survival was measured in months from diagnosis to either death or the end of the observation period. Patients who died within 30 days of diagnosis were given a survival time of 1 month. For patients who lived for ≥1 month, survival was measured in completed months. Correlation analyses were performed using the Spearman r correlation test, and OS was analysed by the Kaplan‐Meier estimator. Subgroup comparisons of categorical and continuous values were calculated using Fisher's exact test and Mann‐Whitney test, respectively. *p*‐Values <0.05 were considered significant.

## 3 RESULTS

### 3.1 Patient characteristics

Baseline data and laboratory values of the 189 patients included in the study are presented in Table 1. Median age was 58 years (range 19–75 years), with male patients making up 60.3% of the patient cohort. The median CRP value was 29 mg/dL (range 1–424 mg/dL), and the median serum albumin level was 39 g/L (range 21–52 g/L). 68 patients received allo‐HSCT, compared to eight patients receiving auto‐HSCT, while 113 patients did not receive any transplant. The median survival for the study cohort was 27 months.

**TABLE 1:** Population demographics and characteristics.

Characteristics	All patients
Number of patients	189
Age (years)	58 (19–75)
Gender, *n* (%)	
Male	114 (60.3%)
Female	75 (39.7%)
Hb (g/L)	9.4 (3.8–15.4)
WBC (x10^9^/L)	10.5 (0.2–660)
Platelets (x10^9^/L)	67 (5–1997)
CRP (mg/L)	29 (1–424)
Albumin (g/L)	39 (21–52)
LDH (U/L)	383 (129–6123)
Creatinine (*µ*mol/L)	75 (40–206)
Transplantation, *n* (%)	
Autologous	8 (4.2%)
Allogeneic	68 (36%)
No transplantation or unknown	113 (59.8%)
CR, *n* (%)	129 (68.3%)
AML FAB classification, *n* (%)	
M0^a^	8 (4.2%)
M1^b^	27 (14.3%)
M2	22 (11.6%)
M4^c^	36 (19.0%)
M5	26 (13.8%)
M6	1 (0.5%)
M7	1 (0.5%)
Unclassified^d^	68 (36.0%)
*FLT3* mutation^e^, *n* (%)	
Yes	49 (25.9%)
No	120 (63.5%)
Unknown	20 (10.6%)
*NPM1* mutation, *n* (%)	
Yes	38 (20.1%)
No	98 (51.9%)
Unknown	53 (28.0%)
Cytogenetics, *n* (%)	
46, XX/46, XY	117 (61.9%)
−7	10 (5.3%)
−5	2 (1.1%)
+8	10 (5.3%)
Inv(16)/t(16:16)	15 (7.9%)
t(9:11)	3 (1.6%)
t(6:11)	2 (1.1%)
inv(3)/‐3	2 (1.1%)
+13	3 (1.6%)
Complex caryotype^f^	12 (6.3%)
Other^g^	5 (2.6%)
No data	8 (4.2%)
ELN risk group, *n* (%)	
Adverse	28 (14.8%)
Intermediate	124 (65.6%)
Favourable	32 (16.9%)
Unclassified	5 (2.6%)

Patient demographics among 189 AML patients aged 18 years or older receiving induction treatment at Haukeland University Hospital between 2001 and 2021. Age and selected laboratory results are shown in median values and highest and lowest values in parenthesis. Gender, transplantation, remission status, FAB classification, mutation status, cytogenetics and ELN group are shown in numeric distribution with percentages in parenthesis.

Abbreviations: AML FAB, French American British classification group for Acute Myeloid Leukaemia; CR, complete remission following first induction; CRP, C‐reactive protein; ELN, European Leukaemia Network; FLT3, FMS‐like tyrosine kinase 3 gene; Hb, haemoglobin; ITD, internal tandem duplications; LDH, lactate dehydrogenase; NPM1, nucleophosmin 1 gene; TKD, tyrosine kinase domain; WBC, white blood cell count.

^a^ Includes AML M0, M0/M1.

^b^ Includes AML M1, M1/M2.

^c^ Includes AML M4, M4eos, M4/M5.

^d^ Includes AML, MDS‐AML, KML‐AML, Myeloid Sarcoma, AML‐RAEB/T.

^e^ Includes both TKD and ITD mutations.

^f^ Presence of three or more structural variants in the absence of recurrent genetic abnormalities [42].

^g^ Not specified recurrent genetic abnormality.

### 3.2 Correlation analysis of laboratory data at the time of diagnosis

When correlating serum levels of CRP to serum levels of albumin, CRP levels demonstrated a significant negative correlation to albumin, a near‐perfect positive correlation to CAR, and a positive correlation to WBC and LDH. The albumin levels correlated significantly negatively with CAR, and weakly positively with Hb values.

**FIGURE 2:**
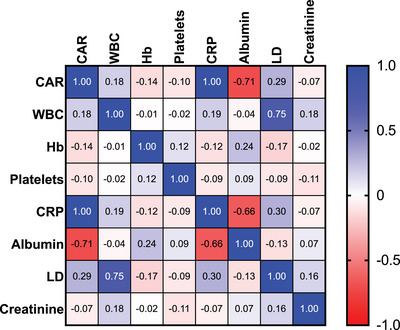
Spearman r correlation matrix showing a correlation between selected laboratory values and CAR values among 189 AML patients aged ≥ 18 years receiving induction treatment at Haukeland University Hospital between 2001 and 2021. CAR: CRP‐albumin ratio. WBC: white blood cell count. Hb: haemoglobin. S‐LD: serum lactate dehydrogenase.

LDH showed a positive correlation with CAR and WBC, and a negative correlation with Hb. Creatinine had a negative correlation with WBC, and WBC had a positive correlation with CAR. A complete correlation overview is given in Figure 2. All mentioned correlations had a *p*‐value <0.05.

*NPM1* mutated patients as a group had higher levels of CRP and WBC, lower levels of albumin and a larger proportion of patients classified with a high CAR compared to *NPM1*‐wild type patients at the time of diagnosis (Table 2). For *FLT3* internal tandem duplication (ITD) or tyrosine kinase domain (TKD) mutated patients, we saw higher levels of CRP, and WBC and lower albumin levels at the time of diagnosis compared to *FLT3*‐wild type patients (Table 3)

**TABLE 2:** NPM1 status, lab values, GPS and CAR.

Characteristic	*NPM1* mutated, n = 38	*NPM1* wild type, n = 98	*p*‐value
WBC (x10^9^/L)	38 (0.6–660)	11 (0.2–247)	0.018*
CRP (mg/L)	58 (4–294)	24 (1–419)	0.009*
Albumin (g/L)	37 (21–47)	39 (23–52)	0.015*
GPS0/1, n, (%)	28 (74%)	87 (81%)	0.37
GPS2, n, (%)	10 (24%)	21 (19%)	
High CAR, n, (%)	26 (68%)	42 (43%)	0.012*
Low CAR, n, (%)	12 (32%)	56 (57%)	

Distribution of selected lab parameters, GPS and CAR between mutated and wild‐type *NPM1* genes in 126 adult AML patients who received induction treatment. Lab values are shown in median values with highest and lowest in parenthesis, and GPS and CAR are shown in numeric distribution with percentages in parenthesis.

Abbreviations: AML, acute Myeloid Leukaemia; CAR, CRP albumin ratio; CRP, C‐reactive protein; GPS, Glasgow Prognostic Score; *NPM1*, nucleophosmin 1; WBC, white blood cell count.

**TABLE 3:** FLT3 status, lab values, Glasgow Prognostic Score (GPS) and C‐reactive protein‐albumin ratio (CAR).

Characteristic	*FLT3* mutated, *n* = 49	*FLT3* wild type, *n* = 120	*p*‐value
WBC (x10^9^/L)	49 (0.2–660)	8 (0.2–131)	<0.0001*
CRP (mg/L)	50 (1–276)	24 (1–424)	0.007*
Albumin (g/L)	36 (21–52)	39 (24–50)	0.0016*
GPS0/1, *n* (%)	33 (67%)	96 (80%)	0.10
GPS2, *n* (%)	16 (33%)	24 (20%)	
High CAR, *n* (%)	31 (63%)	56 (47%)	0.06
Low CAR, *n* (%)	18 (37%)	64 (53%)	

Distribution of selected lab parameters, GPS and CAR between mutated and wild‐type NPM1 genes in 169 adult AML patients who received induction treatment. Lab values are shown in median values with highest and lowest in parenthesis, and GPS and CAR are shown in numeric distribution with percentages in parenthesis.

Abbreviations: AML, acute Myeloid Leukaemia; CAR, CRP albumin ratio; CRP, C‐reactive protein; *FLT3*, FMS‐like tyrosine kinase 3; GPS, Glasgow Prognostic Score; WBC, white blood cell count.

### 3.3 Patient outcome was associated with age, ELN risk group and remission status

**FIGURE 3:**
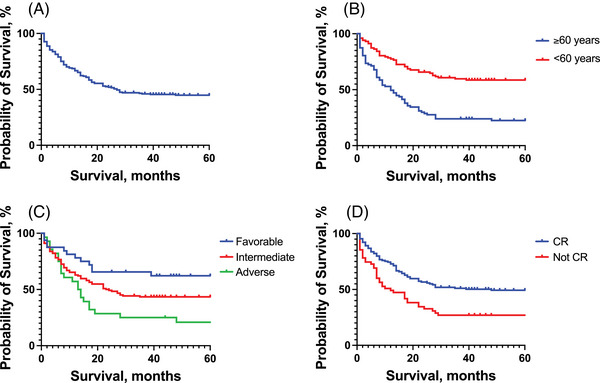
Kaplan‐Meier curves showing survival in 189 adult AML patients receiving induction treatment at HUS between 2001 and 2021. (A) Survival in the study cohort with a median survival of 27 months. (B) Survival among patients aged <60 years at the time of diagnosis compared to patients ≥ 60 years (OS > 50% alive vs. 12 months, *p* < 0.001, log‐rank test). (C) Survival distribution in different European Leukaemia Network (ELN) risk groups (OS > 50% alive vs. 24 vs. 14 months in favourable, intermediate, and adverse, respectively, *p* < 0.005, log‐rank test with the significant trend, *p* < 0.005, log‐rank test for trend). (D) Survival among patients achieving CR compared to those who did not (OS 48 vs. 12 months, *p* < 0.0005, log‐rank test). AML: acute myeloid leukaemia. HUS: Haukeland University Hospital. CR: complete haematological remission after the first induction cycle.

The median survival for the entire patient cohort was 27 months (Figure 3A). Note that, 102 out of the 189 AML patients included in the study were younger than 60 years old at the time of diagnosis.

When comparing patients older than 60 years at the time of diagnosis with patients younger than 60 years old, there was a significant difference in OS in favour of younger patients (OS > 50% at the end of the observation period versus 12 months; *p* < 0.0001, Log‐rank test) (Figure 3B).

Based on cytogenetic data and the mutation status of *NPM1* and *FLT3*, all patients were classified according to the ELN prognostic classification system [4]. Thirty‐two, 124 and 28 patients were classified as favourable, intermediate, or adverse ELN risk scores, respectively. There was a significant difference in OS based on ELN risk stratifications, with a median survival of 14 months versus 24 months versus >50% still alive in adverse, intermediate and favourable risk groups, respectively (*p* < 0.005, Log‐rank test; significant trend; *p* < 0.005, Logrank test for trend) (Figure 3C).

Receiving induction treatment was one of the inclusion criteria in our study. Complete haematological remission (CR) after first induction treatment was registered as stated in the patient journal, based on the accepted definition of < 5% blasts in the bone marrow smear, and CR data were available for 184 out of 189 patients. Note that, 129 patients achieved CR after one induction cycle, while 55 did not. OS was significantly greater among patients who achieved CR after the first induction compared to those who did not (OS 48 vs. 12 months; *p* < 0.0005, Log‐rank test) (Figure 3D). These findings are consistent with well‐established prognostic parameters for AML [1, 2].

### 3.4 Survival analysis based on isolated CRP and albumin levels

**FIGURE 4:**
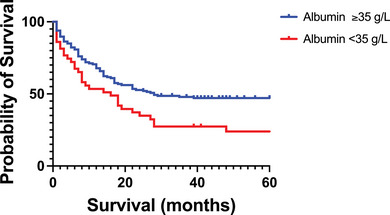
Kaplan‐Meier curve showing statistically significant superior survival among AML patients with a normal albumin level at the time of diagnosis compared to patients with an abnormal low albumin value at the time of diagnosis (OS 28 vs. 16 months, *p* < 0.02, log‐rank test). AML: acute myeloid leukaemia.

Serum CRP and albumin were available for all 189 patients. Patients with normal albumin levels had a significantly superior prognosis compared to patients with hypoalbuminemia (OS 28 vs. 16 months; *p* < 0.02, Log‐rank test) (Figure 4). There was no difference in survival between patients with a CRP level >10 compared to patients with a normal CRP level (OS 25 vs. 21 months; *p* = 0.38, Log‐rank test).

### 3.5 Survival analysis, based on CAR

Serum CRP and albumin levels were available for 189 AML patients at the time of diagnosis. Based on the CAR, median ratio of 0.82, the patients were divided into two groups according to CAR values: a high CAR group (95 patients, CAR ≥ 0.82), and a low CAR group (94 patients, CAR < 0.82) (Table 4). No significant difference in median survival between the two groups was observed. Further sub‐analysis on age, gender, ELN risk group or remission status did not alter the results (*data not shown*).

**TABLE 4:** Patient characteristics, C‐reactive protein (CRP)‐albumin ratio.

Characteristics	Low CAR (<0.82)	High CAR (≥0.82)	*p*‐value^h^
Number of patients	94	95	*>0.99*
Age (years)	60 (19–75)	56 (19–75)	*0.42*
Gender, *n* (%)			
Male	58 (61.7%)	56 (58.9%)	*0.77*
Female	36 (38.3%)	39 (41.1%)	
Hb (g/L)	9.6 (4.1–15.1)	9.3 (3.8–15.4)	*0.66*
WBC (x10^9^/L)	7.7 (0.7–378)	17.8 (0.2–660)	0.014*
Platelets (x10^9^/L)	76 (5–1997)	51 (5–365)	*0.25*
CRP (mg/L)	9 (1–31)	85 (27–424)	<0.0001*
Albumin (g/L)	42 (33–52)	35 (21–45)	<0.0001*
LDH (U/L)	329 (129–6123)	452 (146–2948)	0.008*
Creatinine (µmol/L)	76 (48–159)	75 (40–206)	*0.21*
Transplantation, *n* (%)			
Autologous	3 (3.2%)	5 (5.3%)	*0.72*
Allogeneic	34 (36.2%)	34 (35.8%)	*0.39*
None or unknown	57 (60.6%)	56 (58.9%)	*0.88*
CR, *n* (%)	63 (67.0%)	66 (69.5%)	*0.76*
AML FAB classification, *n* (%)			
M0^a^	4 (4.3%)	4 (4.2%)	*>0.99*
M1^b^	14 (14.9%)	13 (13.7%)	*0.84*
M2	11 (11.7%)	11 (11.6%)	*>0.99*
M4^c^	12 (12.8%)	24 (25.3%)	0.04*
M5	9 (9.6%)	17 (17.9%)	*0.14*
M6	1 (1.1%)	0 (0.0%)	*0.50*
M7	0 (0%)	1 (1.1%)	*>0.99*
Unclassified^d^	43 (45.7%)	25 (26.3%)	0.006*
*FLT3* mutation^e^, *n* (%)			
Yes	18 (19.1%)	31 (32.6%)	0.046*
No	64 (68.1%)	56 (58.9%)	*0.23*
Unknown	12 (12.8%)	8 (8.4%)	*0.36*
*NPM1* mutation, *n* (%)			
Yes	12 (12.8%)	26 (27.4%)	0.018*
No	55 (58.5%)	42 (44.2%)	*0.059*
Unknown	27 (28.7%)	27 (28.4%)	*>0.99*
Cytogenetics			
46, XX/46, XY	53 (56.4%)	64 (67.4%)	*0.14*
−7	6 (6.4%)	4 (4.2%)	*0.54*
−5	0 (0.0%)	2 (2.1%)	*0.50*
+8	7 (7.4%)	3 (3.2%)	*0.21*
Inv(16)/t(16:16)	10 (10.6%)	5 (5.3%)	*0.19*
t(9:11)	0 (0.0%)	3 (3.2%)	*0.25*
t(6:11)	0 (0.0%)	2 (2.1%)	*0.50*
inv(3)/‐3	2 (2.1%)	0 (0.0%)	*0.25*
13	3 (3.2%)	0 (0.0%)	*0.12*
Complex caryotype^f^	5 (5.3%)	7 (7.4%)	*0.77*
Other^g^	3 (3.2%)	2 (2.1%)	*0.68*
No data	5 (5.3%)	3 (3.2%)	*0.50*
ELN risk group, *n* (%)			
Adverse	13 (13.8%)	15 (15.8%)	*0.83*
Intermediate	60 (63.8%)	64 (67.4%)	*0.65*
Favourable	17 (18.1%)	15 (15.8%)	*0.70*
Unclassified	4 (4.3%)	1 (1.1%)	*0.21*

Patient demographics among 189 AML patients aged 18 years or older receiving induction treatment at Haukeland University Hospital between 2001 and 2021, were divided into a CAR value over or equal to 0.82 and a CAR value under 0.82. Age and selected laboratory results are shown in median values and highest and lowest values in parenthesis. Gender, transplantation, remission status, FAB classification, mutation status, cytogenetics and ELN group are shown in numerical distribution with percentages in parenthesis.

Abbreviations: AML FAB, French American British classification group for Acute Myeloid Leukaemia; CAR, CRP‐albumin ratio; CR, complete remission following first induction; CRP, C‐reactive protein; ELN, European Leukaemia Network; FLT3, FMS‐like tyrosine kinase 3 gene; Hb, haemoglobin; ITD, internal tandem duplications.; LDH, lactate dehydrogenase; NPM1, nucleophosmin 1 gene; TKD, tyrosine kinase domain; WBC: White blood cell count.

^a^ Includes AML M0, M0/M1.

^b^ Includes AML M1, M1/M2.

^c^ Includes AML M4, M4eos, M4/M5.

^d^ Includes AML, MDS‐AML, Myeloid Sarcoma, AML‐RAEB/T.

^e^ Includes both TKD and ITD mutations.

^f^ Presence of three or more structural variants in the absence of recurrent genetic abnormalities [42].

^g^ Not specified recurrent genetic abnormality.

^h^ *p*‐Values were calculated using the Mann‐Whitney U test for continuous variables, and the Fisher's exact test for categorical variables. *p*‐Values <0.05 are considered statistically significant.

### 3.6 Survival analysis based on GPS and mGPS

Using the serum values of CRP and albumin, each patient could be classified according to the GPS. Both the original GPS and the mGPS were used, and patients were put into a GPS/mGPS of 0, 1 and 2, where 0 is assumed to predict the best prognosis and 2 is assumed to predict the worst prognosis [10]. In our study, patient distribution into mGPS and GPS groups was identical as none of the hypoalbuminemia patients (i.e. albumin <35 g/L) had a CRP value <10 mg/L. Since GPS2 and mGPS2 are scored by the same criteria (CRP ≥10 mg/L and albumin <35 g/L), GPS1 and mGPS1 also remained identical.

**FIGURE 5:**
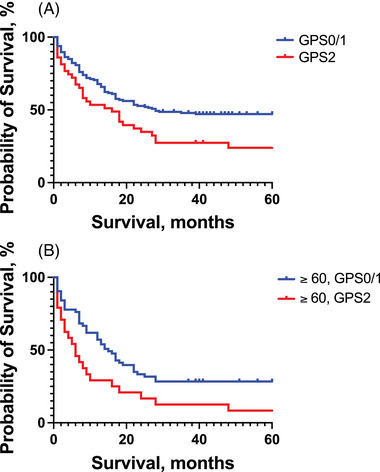
Kaplan‐Meier curves comparing survival measured in months between 189 adult AML patients receiving induction treatment at Haukeland University Hospital between 2001 and 2021 with different Glasgow Prognostic Scores. GPS0 and GPS1 were compared as one group to GPS2. (A) Kaplan‐Meier curve showing superior survival among patients with a GPS0 or 1 compared to GPS2 among all patients (OS 28 vs. 16 months, *p* < 0.02, log‐rank test). (B) Kaplan‐Meier curve showing superior survival with a GPS0 or 1 compared to GPS 2 among patients ≥60 years at the time of diagnosis (OS 15 vs. 6 months, *p* < 0.05, log‐rank test). GPS: Glasgow Prognostic Score. AML: acute myeloid leukaemia.

Fifty‐five, 91 and 43 patients had a GPS of 0, 1 and 2, respectively (Table 5). There was a significant difference in OS between the three groups in favour of GPS0 and GPS1 compared to GPS2 with a median survival of 25, 39 and 16 months, respectively (*p* = 0.048, Log‐rank test). There was, however, no difference between survival in the GPS0 and GPS1 group (25 vs. 39 months, *p* = 0.63. Log‐rank test), thus no significant trend between a GPS of 0, 1 and 2 in our study (no significant trend, *p* = 0.11, Logrank test for trend). Because of this we merged the GPS0 and GPS1 groups in further analysis, regarding them as one group and compared them with patients belonging to the GPS2 group. Among 146 patients in GPS0/GPS1 and 43 patients in GPS2, there was a significant difference in survival with a median survival of 28 months in the GPS0/1 group compared to 16 months in the GPS2 group (*p* = 0.015, Log‐rank test) (Figure 5A).

**TABLE 5:** Patient characteristics, Glasgow Prognostic Score.

Characteristics	GPS0	GPS1	GPS0/1	GPS2	*p*‐value^h^
Number of patients	55	91	146	43	<0.0001*
Age (years)	58 (19–75)	55 (19–74)	56 (19–75)	60 (33–75)	*0.070*
Gender, *n* (%)					
Male	36 (65.5%)	53 (58.2%)	89 (61.0%)	25 (58.1%)	*0.86*
Female	19 (34.5%)	38 (41.8%)	57 (39.0%)	18 (41.9%)	*0.86*
Hb (g/L)	9.9 (6.2–15.1)	9.2 (4.1–15.4)	9.6 (4.1–15.4)	9.1 (3.8–12.5)	*0.058*
WBC (x10^9^/L)	7 (0.7–378)	16.4 (0.8–286)	9.8 (0.7–378)	14.8 (0.2–660)	*0.92*
Platelets (x10^9^/L)	47 (5–1997)	82 (11–365)	80 (5–1997)	44 (5–355)	0.0098*
CRP (mg/L)	4 (1‐10)	36 (11–281)	19 (1–281)	109 (14–424)	<0.0001*
Albumin (g/L)	43 (35–50)	39 (35–52)	40 (35–52)	30 (21–34)	<0.0001*
LDH (U/L)	283 (129–2659)	436 (134–6123)	374 (129–6123)	466 (146–2948)	*0.12*
Creatinine (µ*m*ol/L)	79 (56–157)	73 (40–206)	75 (40–206)	75 (41–177)	*0.67*
Transplantation, *n* (%)					
Autologous	2 (3.6%)	4 (4.4%)	6 (4.1%)	2 (4.7%)	*>0.99*
Allogeneic	18 (32.7%)	37 (40.7%)	55 (37.7%)	13 (30.2%)	*0.47*
None or unknown	35 (63.6%)	50 (54.9%)	85 (58.2%)	28 (65.1%)	*0.48*
CR, *n* (%)	39 (70.9%)	65 (71.4%)	104 (71.2%)	25 (58.1%)	*0.14*
AML FAB, *n* (%)					
M0^a^	3 (5.5%)	0 (0.0%)	3 (2.1%)	4 (9.3%)	0.048*
M1^b^	7 (12.7%)	15 (16.5%)	22 (15.1%)	6 (14.0%)	*>0.99*
M2	8 (14.5%)	12 (13.2%)	20 (13.7%)	2 (4.7%)	*0.17*
M4^c^	8 (14.5%)	14 (15.4%)	22 (15.1%)	14 (32.6%)	0.015*
M5	5 (9.1%)	18 (19.8%)	23 (15.8%)	3 (7.0%)	*0.21*
M6	0 (0%)	1 (1.1%)	1 (0.7%)	0 (0%)	*>0.99*
M7	0 (0%)	1 (1.1%)	1 (0.7%)	0 (0%)	*>0.99*
Unclassified^d^	24 (43.6%)	30 (33.0%)	54 (37.0%)	14 (32.6%)	*0.72*
*FLT3* mutation^e^, *n* (%)					
Yes	5 (9.1%)	28 (30.8%)	33 (22.6%)	16 (37.2%)	*0.074*
No	43 (78.2%)	53 (58.2%)	96 (65.8%)	24 (55.8%)	*0.28*
Unknown	7 (12.7%)	10 (11.0%)	17 (11.6%)	3 (7%)	*0.57*
*NPM1* mutation, *n* (%)					
Yes	4 (7.3%)	24 (26.4%)	28 (19.2%)	10 (23.3%)	*0.67*
No	32 (58.2%)	44 (48.4%)	76 (52.1%)	21 (48.8%)	*0.73*
Unknown	19 (34.5%)	23 (25.3%)	42 (28.8%)	12 (27.9%)	*>0.99*
Cytogenetics					
46, XX/46, XY	30 (54.5%)	57 (62.7%)	87 (59.6%)	30 (69.8%)	*0.28*
−7	2 (3.6%)	8 (8.8%)	10 (6.8%)	0 (0.0%)	*0.12*
−5	0 (0.0%)	0 (0.0%)	0 (0.0%)	2 (4.7%)	*0.055*
+8	6 (10.9%)	3 (3.3%)	9 (6.2%)	1 (2.3%)	*0.46*
Inv(16)/t(16:16)	5 (9.1%)	7 (7.7%)	12 (8.2%)	3 (7.0%)	*>0.99*
t(9:11)	0 (0.0%)	2 (2.2%)	2 (1.4%)	1 (2.3%)	*0.54*
t(6:11)	0 (0.0%)	1 (1.1%)	1 (0.7%)	1 (2.3%)	*0.40*
inv(3)/‐3	1 (1.8%)	1 (1.1%)	2 (1.4%)	0 (0.0%)	*>0.99*
13	3 (5.5%)	0 (0.0%)	3 (2.1%)	0 (0.0%)	*>0.99*
Complex caryotype^f^	3 (5.5%)	6 (6.6%)	9 (6.2%)	3 (7.0%)	*0.74*
Other^g^	1 (1.8)	4 (4.4%)	5 (3.4%)	0 (0.0%)	*0.59*
No data	4 (7.3%)	2 (2.2%)	6 (4.1%)	2 (4.7%)	*>0.99*
ELN risk group, *n* (%)					
Adverse	7 (12.7%)	15 (16.5%)	22 (15.1%)	6 (14.0%)	*>0.99*
Intermediate	37 (67.3%)	56 (61.5%)	93 (63.7%)	31 (72.1%)	*0.36*
Favourable	8 (14.5%)	18 (19.8%)	26 (17.8%)	6 (14.0%)	*0.65*
Unclassified	3 (5.5%)	2 (2.2%)	5 (3.4%)	0 (0.0%)	*0.59*

Patient demographics among 189 AML patients aged ≥ 18 years receiving induction treatment at Haukeland University Hospital between 2001 and 2021, divided into GPS0, 1, 0/1 and 2. Age and laboratory results are shown in median values and highest and lowest values in parenthesis. Gender, transplantation, remission status, FAB classification, mutation status, cytogenetics and ELN group are shown in numeric distribution with percentages in parenthesis.

Abbreviations: AML FAB, French American British classification group for Acute Myeloid Leukaemia; CR, complete remission following first induction; CRP, C‐reactive protein; ELN, European Leukaemia Network; FLT3: FMS‐like tyrosine kinase 3 gene; GPS, Glasgow Prognostic Score; Hb, haemoglobin; ITD, internal tandem duplications; LDH, lactate dehydrogenase; NPM1, nucleophosmin 1 gene; TKD, tyrosine kinase domain; WBC, white blood cell count.

^a^Includes AML M0, M0/M1.

^b^Includes AML M1, M1/M2.

^c^Includes AML M4, M4eos, M4/M5.

^d^Includes AML, MDS‐AML, Myeloid Sarcoma, AML‐RAEB/T.

^e^Includes both TKD and ITD mutations.

^f^Presence of three or more structural variants in the absence of recurrent genetic abnormalities [42].

^g^Not specified recurrent genetic abnormality.

^h^ *p*‐values comparing GPS0/1 with GPS2, using Mann Whitney U test for continuous data and Fischer exact test for categorical data. *p*‐Values < 0.05 are considered statistically significant.

### 3.7 Survival analysis based on GPS/mGPS in older and younger AML patients

Among patients ≥60 years old, a GPS0 or GPS1 score showed superior survival when compared to patients with GPS2. Patients ≥60 years old were divided into three groups; GPS0 (25 patients), GPS1 (38 patients) and GPS2 (24 patients), a statistically significant difference in OS in favour of GPS1 and GPS0 compared to GPS2 was observed (OS 12 vs. 17 vs. 6 months in GPS0, 1 and 2, respectively; *p* = 0.017, Log‐rank test). However, no significant trend (no significant trend; *p* = 0.40, Logrank test for trend). The merged group of GPS0/1 showed statistically significant survival compared to GPS2 (OS 15 vs. 6 months; *p* = 0.020, Log‐rank test) (Figure 5B).

Then we divided 102 patients (<60 years old) according to GPS score into GPS0 (30 patients), GPS1 (53 patients) and GPS2 (19 patients). No significant statistical difference was detected when comparing younger patients that are <60 years of age, between the different GPS groups (OS > 50% vs. > 50% vs. 28 months in GPS0, 1 and 2, respectively; *p* = 0.51, Log‐rank test). The results remained unchanged when merging GPS0 and 1 compared to GPS2 (OS > 50 vs. 28 months in GPS0/1 and GPS2 respectively; *p* = 0.48, Log‐rank test).

### 3.8 Survival analysis based on GPS/mGPS in different gender

All 114 male patients were grouped according to GPS: GPS0 (36 patients), GPS1 (53 patients) and GPS2 (25 patients). We found no difference in OS between the three groups (OS 23 vs. 28 vs. 18 months in GPS0, 1 and 2, respectively; *p* = 0.16, Log‐rank test), neither when comparing GPS0/1 as one group to GPS2 (OS 26 vs. 18 months in GPS0/1 and 2, respectively; *p* = 0.056, Log‐rank test).

We observed no statistically significant difference when comparing female AML patients according to GPS score: GPS0 (19 patients), GPS1 (38 patients) and GPS2 (18 patients). The median OS among females belonging to GPS0, GPS1 and GPS2 was 29, 110 and 12 months, respectively (*p* = 0.19, Log‐rank test). When comparing the GPS0 and 1 as one group versus GPS2 the median OS was 80 and 12 months, respectively (*p* = 0.11, Log‐rank test).

### 3.9 Survival analysis based on GPS/mGPS in different AML risk groups

We found no statistically significant difference in OS within the favourable, intermediate, or adverse ELN risk groups when patients were divided into GPS0, GPS1 and GPS2 groups. The results remained unchanged when comparing GPS0/1 versus GPS2.

Among 32 patients with a favourable ELN risk score, 8, 18 and 6 patients were divided into GPS0, 1 and 2, respectively, with an OS of 112 versus >50% alive versus 14 months in GPS0, 1 and 2, respectively (*p* = 0.16, Log‐rank test). Results did not change when comparing GPS0 and 1 as one group to GPS2 (OS > 50% alive vs. 14 months in GPS0/1 vs. 2, respectively; *p* = 0.058, Log‐rank test).

Patients belonging to the intermediate ELN risk group (124 patients) were then divided according to GPS score; GPS0 (37 patients), GPS1 (56 patients) and GPS2 (31 patients). Between the three groups, OS was 25 versus 84 versus 18 months for GPS0, 1 and 2, respectively (*p* = 0.19, Log‐rank test). Comparing GPS0/1 to GPS2 did not alter statistical significance (OS 29 vs. 18 months for GPS0/1 vs. 2; *p* = 0.080, Log‐rank test).

In the adverse ELN group of 28 patients, the distribution between GPS0, 1 and 2 was seven, 15 and six patients, respectively. OS was 13, 14 and 8 months in GPS0, 1 and 2, respectively (*p* = 0.53, Log‐rank test). GPS0 and GPS1 as one group had a median survival of 14 versus 8 months in the GPS2 group (*p* = 0.26, Log‐rank test).

### 3.10 Remission rates based on GPS/mGPS

We found no statistically significant difference in OS among AML patients who achieved CR, when comparing the different GPS groups or when comparing AML patients who did not achieve CR belonging to either GPS0, GPS1 or GPS2.

Note that, 129 AML achieved CR after the first induction cycle. They were divided according to GPS score; GPS0 (39 patients), GPS1 (65 patients) and GPS2 (25 patients). OS among patients who achieved CR was > 50% alive versus 110 versus 19 months in GPS0, 1 and 2, respectively (*p* = 0.25, Log‐rank test). Comparing GPS0 and 1 as one group versus GPS2 did not change the results (110 vs. 19 months; *p* = 0.10, Log‐rank test).

We then divided AML patients who did not achieve CR into GPS0 (15 patients), GPS1 (24 patients) and GPS2 (16 patients). The median OS were 7, 17 and 8 months in GPS0, 1 and 2, respectively (*p* = 0.27, Log‐rank test). The results did not reach statistical significance when comparing GPS0/GPS1 with GPS2 (13 vs. 8 months, *p* = 0.29, Log‐rank test).

### 3.11 Early deaths

Five of 189 patients had a survival time of less than 30 days from diagnosis, and inductive chemotherapy was initiated in all patients. Median CRP and albumin values for these five patients were 54 mg/L [6–419] and 38 g/L [30–43], respectively, and three patients were classified as high CAR. One, two and two patients were classified as GPS0, GPS1 and GPS2, respectively. One patient died of acute arrhythmia with subsequent cardiac arrest, the remaining four patients died of sepsis with multiorgan failure. Among the four septic patients, one had additional acute pulmonary oedema, and one had additional pulmonary haemorrhage.

## 4 DISCUSSION

Our study represents one of the earliest attempts to examine the prognostic relevance of GPS in AML, a scoring system previously established in various solid cancers [29, 30, 31]. We analysed demographic, clinical and laboratory data collected at the time of AML diagnosis from a patient cohort deemed suitable for intensive chemotherapy. We first demonstrate that our cohort seemed to be representative of the general AML population. This is based on the general demographic data (Table 1), and the fact that the most important prognostic factors, that is, age, the ELN‐classification system and remission achievement, also had significant prognostic impact in our patient cohort (Figure 3). By obtaining crucial laboratory data obtained at the time of diagnosis, we first performed a compression between these data. Interestingly, we found a strong and significant negative correlation between CRP and albumin (Figure 2), indicating that these two central laboratory tests are not independent factors. Traditionally they have been used as biomarkers for inflammation/infection and metabolic status of the patients, respectively, and the correlation links these two hallmarks in a strong association.

Albumin as an independent parameter predicted survival in our cohort (Figure 4). Furthermore, there was a general trend toward decreased levels of albumin and increased levels of inflammatory biochemical parameters such as WBC and CRP among NPM1 mutated patients and patients with FLT3 mutations compared to wild‐type genetic variants (Tables 2 and 3).

We endeavoured to investigate the prognostic significance of the CRP/albumin ratio, a continuous variable, across our entire cohort and within specific patient subgroups. However, our analyses yielded no significant prognostic impact of this ratio, irrespective of gender, age or the ELN risk classification system. Although prior research has indicated associations between the CRP‐albumin ratio and prognosis in both solid cancers and haematological malignancies [9, 12–15, 32, 33], our findings did not reveal any noteworthy prognostic associations in our cohort.

Notably, existing literature has emphasized the strongest prognostic associations with patients exhibiting highly elevated CRP and/or significantly decreased albumin levels. Thus, we opted to explore prognostic impact using a categorical risk score, the GPS, which has demonstrated significant prognostic value in various solid cancer forms [8, 10, 23, 29–31], and to some extent in haematological malignancies, including AML [14, 29, 34]. The GPS categorizes patients into three risk groups (Table 5), with group 2 exhibiting the most inferior prognostic impact. Intriguingly, our analysis uncovered a statistically significant association between overall survival and GPS score. Specifically, patients in the GPS 2 group displayed markedly lower overall survival rates compared to those in groups 0 and 1 (Figure 5A), particularly evident in those aged 60 and above (Figure 5B). These findings underscore the notion that the prognostic significance of CRP and albumin levels in AML appears to be predominantly linked to patients with the highest CRP levels and/or lowest albumin levels. Thus, there appears to be a critical threshold where the impact of CRP and albumin levels becomes clinically relevant, as evidenced by our study's association with patients in the GPS group 2. These observations align with previous studies exploring the effect of GPS scores in solid cancers, where group 2 has consistently been associated with the poorest outcomes and survival rates [8, 10, 24–26, 29, 30].

CRP serves as a biomarker for systemic inflammation [35]. On the other hand, albumin reflects the nutritional and metabolic status of the patient. Hypoalbuminemia often signifies a compromised nutritional status, increased disease burden and higher mortality rates [36]. Moreover, low albumin levels have been associated with an elevated risk of treatment‐related toxicities and poorer tolerance to chemotherapy [37].

Our study indicates that evaluating both CRP and albumin levels at the time of an AML diagnosis provides valuable insights into the inflammatory state, nutritional status and tolerability and response to treatment. Integrating these standard markers into clinical practice aids in risk stratification, treatment decision‐making and prognostication, and can ultimately improve patient care and outcomes for this challenging group of patients.

Our study is subject to some limitations that warrant acknowledgement. First, the inclusion of 189 AML patients inherently constrains statistical power, potentially leading to the oversight of subtle differences between groups, particularly for subgroup analyses. Furthermore, being a single‐centre study, we cannot fully elucidate biases stemming from diagnostic and treatment decisions related to the study cohort. Nonetheless, our findings suggest that the demographic data and prognostic system of our cohort are comparable to the broader patient population. Additionally, our observation period spanning 20 years raises considerations regarding changes in treatment paradigms. While the standard induction treatment for AML remained relatively stable during this period [38], significant advancements in supportive care and consolidation treatments, including allo‐HSCT, have occurred [39]. Notably, the risk classification evolved throughout the study period, particularly highlighted by changes in the ELN classification for FLT3‐mutated patients [40]. Furthermore, the current classification system includes recurrent mutations [4], which were not available for the majority of our study population. Finally, both CRP and albumin levels are dynamic during the course of AML treatment, and alterations in these biomarkers are likely relevant and of interest for prognostic outcomes. In addition, weight alterations during treatment have also been shown to have prognostic relevance [41]. Further, longitudinal studies should evaluate these features of AML therapy. Thus, while acknowledging these limitations, our study provides valuable insights into the AML landscape over the past two decades.

To conclude, patients with a GPS of 2 had significantly inferior survival compared to patients scoring 0 and 1. As a supplement to traditional patient‐related and newer disease‐related factors, using simple, inexpensive and accessible parameters such as the CRP and albumin at the time of diagnosis could aid decision‐making and prognostication in patients with newly diagnosed AML who are considered eligible for induction therapy. Further larger and prospective studies should further evaluate the prognostic impact of CRP and albumin levels in AML.

## AUTHOR CONTRIBUTIONS

We confirm that all authors have contributed to the work in a way that fulfils the criteria for authorship given by the International Committee of Medical Journal Editors (ICMJE).

## CONFLICT OF INTEREST STATEMENT

The authors declare no conflict of interest.

## FUNDING INFORMATION

The authors received no specific funding for this work.

## ETHICS STATEMENT

The authors have confirmed ethical approval statement is not needed for this submission.

## PATIENT CONSENT STATEMENT

Please refer to the main text section “Patient Population and Methods”.

## CLINICAL TRIAL REGISTRATION

The authors have confirmed clinical trial registration is not needed for this submission.

## Data Availability

The data that support the findings of this study are available from the corresponding author upon reasonable request.
